# Quantification of bacterial adhesion to tissue in high-throughput kinetics

**DOI:** 10.1093/biomethods/bpad014

**Published:** 2023-07-26

**Authors:** Nimrod Shteindel, Danielle Gutman, Gil Atzmon, Yoram Gerchman

**Affiliations:** Department of Evolutionary and Environmental Biology, Haifa University, Tivon, Israel; Department of Human Biology, University of Haifa, Haifa 3498838, Israel; Department of Human Biology, University of Haifa, Haifa 3498838, Israel; Department of Evolutionary and Environmental Biology, Haifa University, Tivon, Israel; Institute of Evolution, University of Haifa, Haifa 3498838, Israel; Department of Biology, Oranim College, Kiryat Tivon 3600600, Israel

**Keywords:** kinetics, adhesion of bacteria to tissue, high throughput, *Pseudomonas aeruginosa*

## Abstract

Bacterial adhesion to tissue is the starting point for many pathogenic processes and beneficial interactions. The dynamics and speed of adhesion (minutes) make high-resolution temporal kinetic data important, but this capability is absent from the current toolset. We present a high-throughput method with a second-to-minute kinetic resolution, testing the adhesion of *Pseudomonas aeruginosa* PAO1 wild-type, flagella-, pili-, and quorum-sensing mutants to human embryonic kidney (HEK293) cells. Adhesion rates were in good correlation with HEK293 confluence, and the ways in which various bacterial mutations modified adhesion patterns are in agreement with the published literature. This simple assay can facilitate drug screening and treatment development as well as provide a better understanding of the interactions of pathogenic and probiotic bacteria with tissues, allowing the design of interventions and prevention treatments.

## Introduction

Bacterial interactions, in both pathogenic and probiotic contexts, depend on the bacterial ability to adhere to the host cells [1]. The importance of this process led to the development of multiple methods of study. These include extraction of bacteria from tissue, quantification by plating, and counting colony-forming units [2]; extraction of bacterial DNA and quantification by quantitative PCR [3]; microscopy imaging for counting bacteria after tissue fixation [4], with or without fluorescence dye labeling [5]; fluorescence-activated cell sorting (FACS) after fluorescence labeling of the bacteria [6]; or fluorescence microscopy [7]. Nevertheless, besides direct microscopy of fluorescently labeled bacteria, all the above methods are destructive and therefore preclude kinetic quantification, and when using them, we are likely to miss temporal differences that may be critical to our understanding of the adhesion process. Last, these methods require expensive equipment and/or are labor intensive, adding further complication to screening experiments [8].

We developed a simple method based on [8] to allow rapid high-throughput measurement of bacterial adhesion kinetics to tissue culture. We utilize fluorescently tagged bacteria (here *Pseudomonas aeruginosa* PAO1 expressing Green Fluoresence Protein), and follow the kinetic of its adhesion to human cells (human embryonic kidney; HEK293) in a multitier 96-well plate using a plate reader capable of measuring bottom fluorescence. To mask the fluorescence emitted by nonadhering, planktonic bacteria, we add a dye (Allura Red) that absorbs fluorescence at both the excitation and emission wavelengths of the fluorophore, masking nonbottom-adhered bacteria.

## Materials and methods

### Instrumentation

This assay requires a plate fluorimeter with bottom-reading capability. Here we use a multimode plate reader (Synergy HT, Biotek, VT, USA). When testing other instruments, we find that filter-based fluorimeters were superior to monochromator-based fluorimeter (Biotek H1), probably because the broader wavelength spectrum typically measured in filter-based systems allows a more sensitive measurement, although this could be model dependent. Fluorescence was measured from the bottom position (excitation 485/20 nm, emission 528/20 nm, gain 60). OD_600nm_ (optical density at 600 nanometer) was measured using the same plate reader in a volume of 100 µl (optical path of 0.3 cm) using a sterile buffer as blank.

### Strains and culture conditions

#### Human cells

HEK293 [9] were chosen as they are easy to cultivate and a very common surface-adhering cell line, with relevance to *P. aeruginosa* ability to infect kidneys [10]. The cells were seeded in a 96-well plate at 5K, 10K, 20K, and 30K cells per well, in Dulbecco’s Modified Eagle medium (Biological Industries, Israel) supplemented with 10% Fetal Bovine Serum (Biological Industries) and 1% Penicillin–Streptomycin antibiotics (Biological Industries), 100 µl per well. The plate was incubated in a CO_2_ incubator with constant humidity at a temperature of 37°C, at 5.5% CO_2_, for 24 h. Shortly before the addition of the bacteria confluence was estimated, and the medium was removed and replaced with 50 µl of Dulbecco’s Phosphate Bufferd Saline (DPBS; Sartorius, Israel, Cat#02-023-1A) for bacterial adherence experiments.

#### Human cells confluence

The confluence of the HEK293 cells was estimated after the 24-h incubation using Nikon Eclipse TS100 (Nikon, USA) inverted microscope equipped with Nikon 10×/0.25 objective. Pictures were taken with an iPhone X (Apple, USA) using an aftermarket adapter with 10X ocular, and the images were analyzed for confluence by eye and by using the Cell and Gene Therapy Catapult web application [11] (https://ct.catapult.org.uk/resources/confluency-tool), gaining very similar results.

### Bacteria

*Pseudomonas aeruginosa* PAO1 (*P. aeruginosa* PAO1) was used as model bacteria. *Pseudomonas aeruginosa* is a common opportunistic pathogen infecting a great variety of plant and animal species including humans [12], and notorious for its adherence to the lung tissue of cystic fibrosis patients [13], severe burns [14], contamination of I.V. catheters and intubation devices [15, 16, respectively), and kidney infection [10]. These bacteria are also common adhesion and biofilm formation models [17]. The w.t. and Δ*flg*F were purchased from the Washington University Manoil laboratory mutant library [18]; the Δ*pqs*A and Δ*las*I mutants [19] were kindly provided by Professor Miguel Camara of Nottingham University; the Δ*fli*C and Δ*pil*A mutants [20] were kindly provided by Professor Martin Welch of Cambridge University. All *P. aeruginosa* strains tested were transformed by electroporation with the pMRP9-1 plasmid, for constitutive expression of GFPmut2 and Carbenicillin resistance [21], kindly provided by Professor Ehud Banin of the Bar Ilan University.

### Preparation of bacteria for adherence experiments

Before each experiment, bacteria were cultivated in 6 ml of M9 medium complemented with 0.4% glucose and 200 μg/ml Carbenicillin (all from Merc-Sigma, Israel) in 18 mm glass test tubes for 18 h at 37°C, shaking at 120 round per minute (RPM).

Bacteria were collected by centrifugation (5000 g, 1 min, 25°C), washed twice in DPBS without calcium and magnesium, and re-suspended in DPBS (as above), diluted to OD_600nm_ = 0.1 (measured in 100 µl/well in a 96-well plate), and supplemented with 1.6 mg/ml Allura-red AC dye from a stock solution of 100 mg/ml (Dye bought from Sigma, Israel; National Center for Biotechnology Information, PubChem Compound Summary for CID 33258, Allura Red AC. https://pubchem.ncbi.nlm.nih.gov/compound/Allura-Red-AC. Accessed July 31, 2023) [8]. For total fluorescence quantification, *P. aeruginosa* was diluted to OD_600nm_ = 0.05 with DPBS as measured for 100 µl/well in a 96-well plate (equivalent to 0.15 in 1 cm cuvette), fluorescence was measured, and results were used to normalize adhesion results.

### Measuring adhesion kinetics to HEK293 cells in various confluences

Fifty microliters of *P. aeruginosa* PAO1 w.t. was added to each well of the plate containing HEK293 cells in the indicated confluences, resulting in a final dye concentration of 0.8 mg/ml and final bacterial culture density of OD_600nm_ = 0.05. The plate was immediately loaded onto the plate reader, and bottom fluorescence was read every 30 s for 1 h. Each treatment was carried out in 12 replicate wells. An 8-channel multichannel pipettor was used for speed and the whole plate was loaded in <1 min.

### Kinetic adhesion of *P. aeruginosa* PAO1 mutants

HEK293 cells were cultivated (as in “Human cells” section) to a confluence of 20 000 cells per well. The various *P. aeruginosa* PAO1 mutant cultures were cultivated and prepared as described above and added in 50 µl, six duplicate wells per mutant culture. Adhesion measurements were carried out as described earlier, taking a measurement every 60 s for 1 h. After the kinetics measurements, the plate was gently emptied and the liquid was replaced with 100 µl of sterile DPBS supplemented with 0.8 mg/ml dye, measuring bottom fluorescence once more to observe adhesion in the absence of unattached bacteria.

### Normalizing fluorescence

Different *P. aeruginosa* mutants were found to have a different base fluorescence, even at similar bacterial density, probably due to differences in GFP expression. To correct for this difference, we normalized the kinetic and post-kinetic fluorescence measurements. To this end, after growth, washing, and dilution the bacteria to OD_600nm _<0.5 (see “Preparation of bacteria for adherence experiments” section), 100 µl of each bacterial strain was measured for OD_600 nm_ (as a measure of bacterial density) and for fluorescence (without dye, to average the whole population). The OD and fluoresence measurements were used to calculate the normalization fluorescence as detailed in Equation (1).

Calculation of normalized fluorescence


(1) Normalized fluorescence= Fluoresence (with dye) Base fluoresence (no dye) OD600nm(no dye)

### Differentiating total and strongly adhered bacteria

After kinetics, in order to differentiate between strongly and loosely adhering bacteria, we removed the liquid from each well by pipetting and refilled the wells with 100 µl of DPBS supplemented with 0.8 mg/ml dye for a uniform background. Next, bottom fluorescence was measured once more to assess adhesion in the absence of nonadhering or loosely adhering bacteria.

### Statistical analysis

Statistical analysis was performed using SPSS (IBM SPSS Statistics for Windows, Version 24.0. Armonk, NY: IBM Corp.), and post hoc tests used Tukey LSD.

## Results

**Figure 1.**
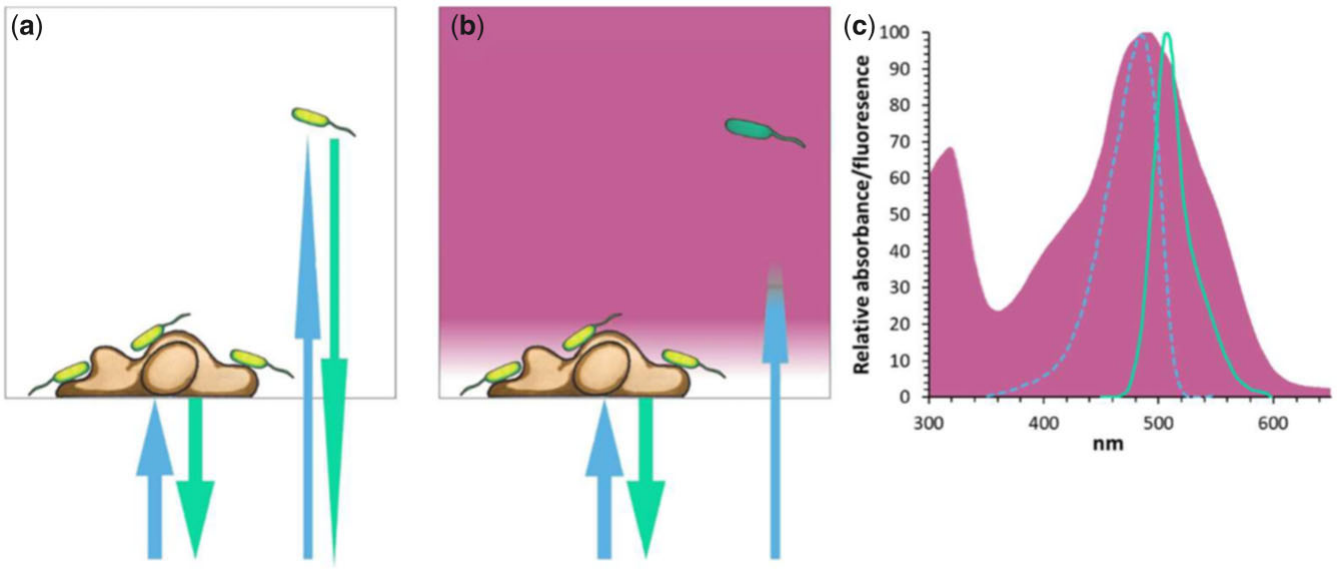
Illustration of the method for quantifying bacterial adhesion to HEK293 cells in the absence (**a**) and presence (**b**) of dye. Bacteria are illustrated in green (as they express GFP) and HEK293 cells in orange. In the absence of dye (**a**) the fluorescence of both adhering and suspended bacteria is read, while with dye (**b**) the GFP excitation and emission of light is decaying due to absorption by the dye molecules. (**c**) Absorbance spectrum of Allura-red AC (the dye; red fill), excitation (cyan), and emission (green) spectrum of GFP. The addition of dye blocks excitation light from reaching suspended bacteria (b), optically isolating the signal produced by adhering bacteria (Adapted with permission from Shteindel et al. [8] and from Shteindel and Gerchman [21]).

The method described here is illustrated in Fig. 1. Briefly, the human cells are attached to the bottom of the wells, and fluorescence-tagged bacteria are added to the liquid above the cells (Fig. 1a). Allura-red, a nontoxic dye with strong optical absorbance at the excitation and emission wavelength of GFP (Fig. 1c), is added with bacteria to quench fluorescence from planktonic nonadhering bacteria that are further away from the human cells (Fig. 1b).

**Figure 2.**
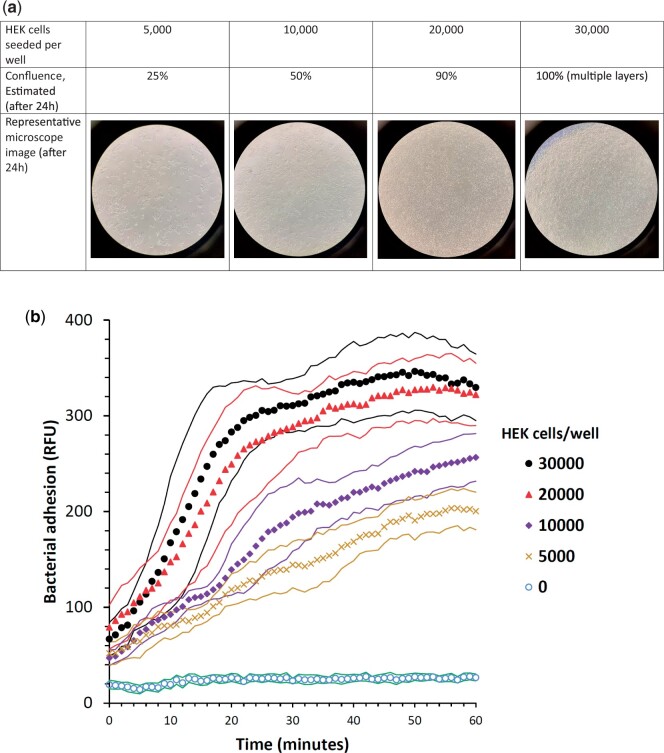
Adhesion of *P. aeruginosa* PAO1 w.t. to HEK293 cells in various cell confluences. (**a**) Number of seeded HEK293 cells per well, estimated confluence, and representative microscopy images. (**b**) Adhesion kinetics of the GFP-labeled *P. aeruginosa* for different HEK293 concentrations/confluences; dots stand for measurement times, flanking thin lines are for ±1 SD, *n* = 12 per treatment.

**Figure 3.**
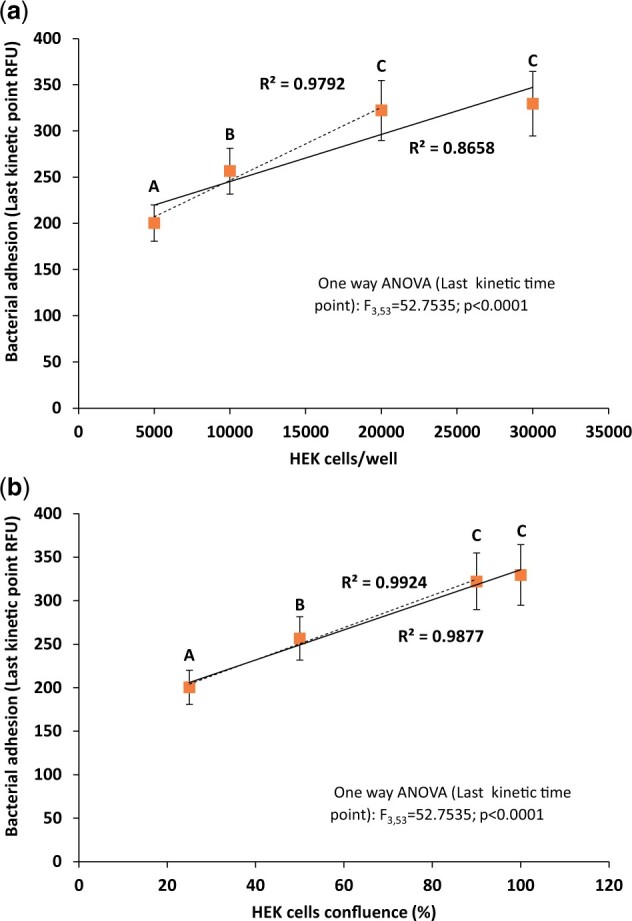
Correlation between HEK293 cells concentration/confluences and *P. aeruginosa* PAO1 w.t. adhesion. (**a**) Last kinetics point adhesion in different HEK293 concentrations; *n* = 12 per treatment, error bars stand for ±1 SD. (**b**) The same data as in (a) but fitted against HEK293 confluence (see Fig. 2a). For both (a) and (b), the data were fit to linear model for the whole data (smooth line) and for the three first HEK cells concentrations/confluencies (dashed line). Capital letters above symbols denote statistically significant different results as determined by One-way ANOVA with Tukey post-hoc test, *P* < 0.01.

Seeding different numbers of HEK293 cells into the wells resulted in different and correlated confluency (Fig. 2a). Measuring adherence of *P. aeruginosa* PAO1 to the different HEK293 cell numbers revealed a strong effect; with very small adherence without HEK293 cells (Fig. 2b, “0 HEK cells”) and higher cell concentration/confluence resulting in faster and higher final adherence of the bacteria (Fig. 2b). When correlating the last kinetic point fluorescence with HEK293 cell concentration, a very good linear correlation was evident up to 20 000 cells (Fig. 3a, dashed curve fit; *R*^2^ = 0.9792). Having 30 000 cells did not improve the bacterial adherence (compare Figs 2b and 3a, 20 000 and 30 000 HEK293 cells/well), and regression quality degraded when the 30 000 cells data were included (Fig. 3a, dashed and smooth regression lines; *R*^2^ = 0.8658 and 0.9792, with and without 30 000 HEK293 cells, respectively). When correlating bacterial adherence to HEK293 confluence (Fig. 3b, dashed and smooth regression lines), the difference between correlations was negligible (*R*^2^ = 0.9877 and 0.9924, respectively), suggesting bacteria prefer to adhere to the exposed cell/tissue surface rather than between the cells/the exposed plastic surface.

**Figure 4.**
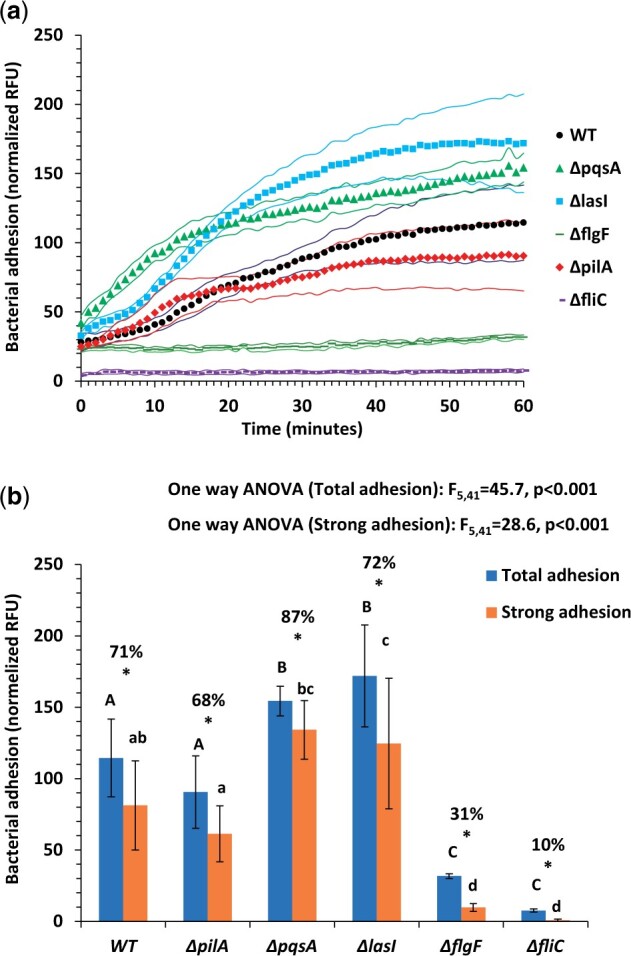
Adhesion of various *P. aeruginosa* mutants to HEK293 at 20 000 cells per well (**a**). Adhesion kinetics of the various mutants; dots stand for measurement times, flanking thin lines are for ±1 SD. (**b**) Total adhesion (last kinetic time point; Blue columns) and strong adhesion (left after removal of loosely adhering bacteria; Orange columns). *n* = 12 for w.t. *P. aeruginosa*, *n* = 6 for mutant; error bars stand for ±1 SD. Different capital letters (**b**) above the blue columns represent statistically different groups for total adhesion and different lower case letters above the orange columns represent statistically different groups for strong adhesion (*P* < 0.05 by one-way ANOVA with post-hoc). Asterisks above blue-orange columns indicate a statistically significant difference between total adhesion and strong adhesion for the same mutant (*P* < 0.05 in one-tailed Welch’s *t*-test comparison within each mutant). Percentage above the columns stands for percentage of strong adhesion of total adhesion.

When comparing different mutants (Fig. 4), results fit our current understanding of *P. aeruginosa* adhesion behavior– bacteria readily adhere to eukaryotic cells [22, 23], using the flagellum for initial adhesion to the surface of the cells [24]. This causes flagella mutants (Δ*flg*F and Δ*fli*C) to display no significant adhesion kinetics (Fig. 4a) and low total adherence (Fig. 4a and b). When testing for strong adherence (i.e. after the removal of liquid and un-/loosely adhered bacteria) and total adherence (last kinetic time point, Fig. 4b), these mutants also display low strong adherence (31 and 10%, respectively). The lack of kinetic change in fluorescence also demonstrated that sedimentation is not an issue in this system – the fluorescence of these mutants remains low and constant throughout the kinetic experiment.

Pili mutant (Δ*pil*A) adhesion is similar to that of the wild-type, for both total and strong adhesion, in agreement with the results of [25], showing that pili are not used in tissue adhesion but rather facilitate bacterial binding to hydrophobic surfaces. Both QS mutants (Δ*las*I and Δ*pqs*A) showed statistically higher adherence to the cells (Fig. 4b), in agreement with the published results [26]. Nevertheless, the Δ*las*I shows higher total adherence combined with lesser strong adherence, when compared to the Δ*pqs*A mutant (87% and 72% respectively), a phenomenon worthy of further exploration.

## Discussion

Adherence of bacteria to host cells is an important step in both pathogenic and probiotic interactions. Here we present a method for simple quantification of the kinetics of bacterial adherence to tissue culture, using HEK293 and *P. aeruginosa* as a model. This method can be extended to any surface adhering cell lines (e.g. most epithelial cells as well as many nonmammalian cells) and to many other bacteria [8], as long as they can be fluorescently labeled, by expressing a fluorescence protein, a metabolic dye, or otherwise. Furthermore, this approach relies on commercially available multi-purpose equipment (i.e. bottom fluorescence plate reader, fluorescence-tagged bacteria, and simple dye tuned for the fluorescence tag). This approach can be easily modified for other microorganism–cell pairs (and even other motile cells), adding to our toolset for studying the adherence of microorganisms to tissue. The experiments provide consistent results, in agreement with published literature, making for a good bacterial adhesion kinetics measurement in a high-throughput setting at low labor and material cost. We hope this method drives research into specific tissue adhesion mechanisms and preclinical testing.

## Data Availability

Data are incorporated into the article. raw data will be shared on reasonable request to the corresponding author.
